# Preserved auditory-motor synchronization during finger-tapping to music and metronomes at various tempi in progressive multiple sclerosis

**DOI:** 10.3389/fneur.2025.1547573

**Published:** 2025-05-02

**Authors:** Nele Vanbilsen, Peter Feys, Mattia Rosso, Bart Van Wijmeersch, Daphne Kos, Marc Leman, Sonja A. Kotz, Lousin Moumdjian

**Affiliations:** ^1^Faculty of Rehabilitation Sciences, REVAL Rehabilitation Research Center, University of Hasselt, Diepenbeek, Belgium; ^2^Universitair Multiple Sclerosis Centrum (UMSC) Hasselt-Pelt, Hasselt, Belgium; ^3^Faculty of Arts and Philosophy, IPEM Institute of Psychoacoustics and Electronic Music, University of Ghent, Ghent, Belgium; ^4^Rehabilitation and MS Centre Overpelt, Pelt, Belgium; ^5^National MS Center Melsbroek, Melsbroek, Belgium; ^6^Department of Rehabilitation Sciences, KU Leuven, Leuven, Belgium; ^7^Department of Neuropsychology and Psychopharmacology, Faculty of Psychology and Neuroscience, Maastricht University, Maastricht, Netherlands

**Keywords:** sensorimotor synchronization, progressive multiple sclerosis, music, metronomes, finger-tapping, stability index, EEG

## Abstract

**Background:**

Persons with relapsing and remitting multiple sclerosis (MS) can synchronize steps with music and metronomes at different tempi. However, progressing demyelination, loss of neural connectivity and increased cognitive impairment likely affects how persons with progressive MS (PwPMS) synchronize movements with external beats. We tested how PwPMS tap to music and metronomes at high and low tempi in order to understand auditory-motor capacities behaviorally in PwPMS. Synchronization at brain level was measured using EEG. We aim (1) to investigate whether PwPMS can synchronize taps to various tempi and musical structures (music and metronomes) compared to healthy controls (HCs) (2) to measure neural entrainment to understand the neural basis of synchronization.

**Methods:**

Participants synchronized finger taps to beats in music and metronomes at five tempi: preferred tapping frequency (0%), slow (−8, −4%), and fast (4, 8%). A mixed model analyzed synchronization outcomes, while regression identified clinical factors affecting consistency. Spearman-rank correlations assessed correlations between neural entrainment and behavioral synchronization consistency.

**Results:**

Sixteen HCs and nineteen PwPMS (mean age = 52.42, mean EDSS = 4.24) participated. No significant differences were seen in behavioral and neural synchronization outcomes between PwPMS and HCs across tempi. Behaviorally, synchronization was higher with the metronomes than with music (*p* = 0.01), yet non-significant at neural level. Disability (*p* = 0.02) and manual dexterity (*p* < 0.001) affected synchronization consistency for metronomes, while cognitive impairment affected synchronization consistency for music.

**Conclusion:**

PwPMS show preserved auditory-motor synchronization capacities however influenced by motor and cognitive factors. The study results support considering the use of auditory-motor synchronization for rehabilitation of PwPMS.

## 1 Introduction

MS is a chronic inflammatory and neurodegenerative disease affecting the central nervous system, characterized by demyelination and axonal degeneration (1). With progression of the disease a wide range of impairments can be seen, specifically for walking. Prior research indicated that task-oriented training such as sensorimotor synchronization (2, 3), where a person is asked to walk to beats in music and metronomes might mitigate these impairments. For example, when a person walks to beats in music or metronomes this has a positive effect in predominantly relapsing-and-remitting persons with MS (3). Auditory-motor synchronization can be observed when the rhythm of movements (footsteps/ finger taps) synchronizes with an auditory rhythm (beats). Once the movement and beats synchronize and align in time, during a process of entrainment, they are considered synchronized (4), reducing gait variability (5) and improving gait performance (6). Overall, it has been shown that when walking, for healthy controls (HCs) higher consistent synchronization is seen compared to persons with MS (3). These differences in synchronization consistency may either result from impaired motor responses or impaired temporal predictions. One way to explain the differences in synchronization between healthy participants and persons with MS is the slower processing of auditory stimuli in neurological populations (7), possibly complicating the processing of the rhythmic structure at high tempi. More specifically, for MS, studies have indicated impairments of central auditory processing (8) due to damage along the auditory pathways, altering its integrity. This results in delayed auditory-evoked potentials in MS (9), which, due to progressive loss of neural connectivity, could be more pronounced in persons with progressive MS (PwPMS). Furthermore, in PwPMS more cognitive impairment is seen (10), specifically in terms of information processing speed, likely compromising the temporal processing and generation of an internal representation of the rhythmic structure (11). This could be more prominent when asked to synchronize to higher tempi compared to the preferred baseline frequency and/or complex rhythmical structures as seen in music.

The abovementioned studies investigated auditory-motor synchronization during walking which not only, requires the prediction of sound but also as balance and coordination. To investigate auditory-motor synchronization in a more controlled environment, tapping synchronization tasks have been used (12). However, during tapping, successful synchronization also relies on the generation of an appropriate motor response. MS research has shown that even with minimal disability, synchronization of finger-tapping to an acoustic metronome is less accurate for temporal and spatial aspects compared to HCs (13). However, this study used proxy of synchronization and the task required more complex sequential finger coordination movements, it is still unclear whether auditory-motor synchronization is impaired in PwPMS. In persons with MS with low disability, a low prevalence of upper limb impairment was seen during clinical tests (14). However, a simple finger-tapping task has proven to be an effective method for assessing upper limb dysfunction in PwPMS, even in cases of low disability (15) and involves key motor systems such as the corticospinal tract, cerebellar motor circuits, and proprioceptive pathways (16).

To measure auditory-motor synchronization, research has mainly focused on behavioral measurements using a simple tapping task. Additionally, one can measure entrainment at the neural level. Research has shown that mapping the frequency of the auditory stimulation onto the spectrum of the brain activity is a convenient way to investigate neural processes related to beat processing (17–19). However, to quantify neural entrainment as a dynamic process of phase-alignment to the auditory stimulation (20, 21), the stability index was more recently developed (22) as a measure of the variability of the entrained neural component over time.

To investigate auditory-motor synchronization abilities in PwPMS our study investigated auditory-motor coupling to different tempi (0%: preferred tapping frequency, −8-4%, 4 and 8% of the preferred tapping frequency) to understand whether high tempi impose challenges in the processing of sounds in PwPMS and low tempi could impose additional challenges on motor adaptability. Increments of 4% were chosen based on evidence from previous studies, which suggest that an increase of more than 4% above the preferred frequency engages active cognitive control to maintain synchronization (23). Furthermore, we investigated the impact of disability in PwPMS using a tapping task to beats in music and metronomes. In order to measure neural entrainment, electroencephalography (EEG) was recorded during all tapping conditions. We hypothesized that PwPMS would show lower synchronization consistency to high and low tempi compared to HCs. Additionally, we hypothesized that disability level, manual dexterity and cognitive impairment would impact synchronization consistency in PwPMW. Furthermore, we hypothesized that we would find overall lower synchronization consistencies in PwPMS compared to HCs. Last, we hypothesized that neural entrainment would be less stable in PwPMS compared to HCs.

## 2 Materials and methods

### 2.1 Participants

**Figure 1:**
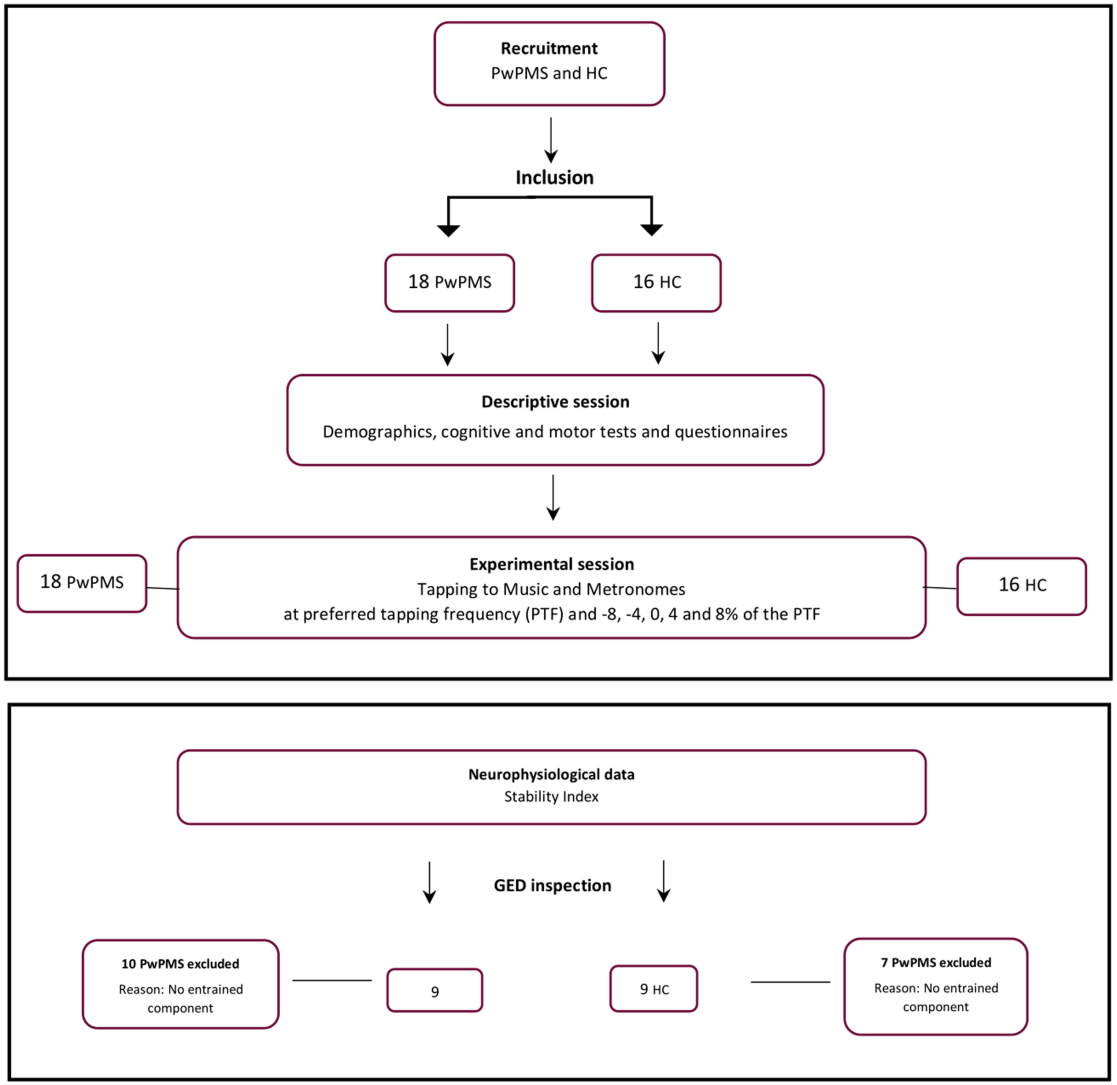
Flowchart illustrating the study selection process, participant flow and experimental procedure. PwPMS- persons with progressive multiple sclerosis, HC- healthy controls.

The experiment was approved by the Medical Ethical Committees of Hasselt University, the National MS Center Melsbroek, and the Noorderhart Rehabilitation and MS Center on January 20, 2021 (B1152020000019) and registered on the clinical.gov website (registration number: NCT04856384). Participants were recruited using study flyers distributed via social media. PwPMS were additionally recruited in the MS centers and advertisements on MS-related social media platforms such as the University MS Center (UMSC) and the Flemish MS association (MS Liga Vlaanderen). Prior to commencing the experimental session, participants agreed to, and signed the informed consent form, after having received detailed information about the experimental session. Inclusion criteria comprised a diagnosis of MS (> 1 year), absence of exacerbation in the preceding month and being older than 18 years and being right-handed as assessed by the Edinburgh Handedness Inventory (24). Exclusion criteria included pregnancy, hearing impairment (assessed by the assessor), or cognitive impairment (assessed by the examiner’s observations) and hindering the understanding of the study instructions. HCs were age-matched to the PwPMS. The same in- and exclusion criteria applied for HC recruitment (Figure 1).

Afterwards, participants were invited for the experiment consisting of one descriptive demographic and clinical information session, and one experimental session.

### 2.2 Session one: descriptive demographic and clinical information

During the first session, we collected demographic information, disease-related data (e.g., EDSS) using patient records in the MS centers when available and assessed musical abilities using the Montreal Battery for Amusia (subscale rhythm) (25). Handedness was assessed using the Edinburgh Handedness Inventory (24) as only right handed participants could be included in the experiment due the EEG analyses Additionally, to assess motor and cognitive functions, standardized tests were employed by the researcher or therapist and patient-reported outcomes were completed by the participant:

#### 2.2.1 Motor functions

Upper extremity functioning was assessed using the Nine-Hole-Peg test (9HPT) for the left and right hand (26). However, our experimental design only utilized right-hand data from the 9HPT, as the tapping experiment was exclusively conducted with the right hand.

#### 2.2.2 Cognitive functions

Participants underwent assessments such as the Buschke Selective Reminding Test (BSRT) (27) for verbal learning and memory, the 7/24 Spatial Recall Test (28) for visual learning and recall, and the Controlled Oral Word Association Test (COWAT) (29) for verbal fluency. The Paced Auditory Serial Addition Test (PASAT) (30) evaluated sustained attention, auditory information processing speed, and flexibility, and the Symbol Digit Modalities Test (SDMT) (31) for information processing speed. The Stroop Color Test (32) examines executive function and inhibitory control.

#### 2.2.3 Self-reported questionnaires

The Hospital Anxiety and Depression Scale (HADS) (33) detects symptoms of depression or anxiety and the Barcelona Music Reward Questionnaire (BMRQ) (34) provided insight into global sensitivity to music reward.

### 2.3 Session two: experimental session

The experimental task consisted of five listening and five tapping synchronization tasks to both music and metronomes while participants were seated comfortably and were asked to sit as still as possible. The listening trials were conducted to untangle the perceptual and the sensorimotor entrained component in the same rhythmic task. Participants were equipped with a 64-channel waveguard original EEG headset (10–10 system, with Ag/AgCl electrodes) and were seated in a comfortable chair in front of a table in a quiet room. To deliver the auditory stimuli, participants were equipped with the DefenderShield air-tube earplugs connected to the D-Jogger (35) system to provide the auditory stimuli.

**Figure 2:**
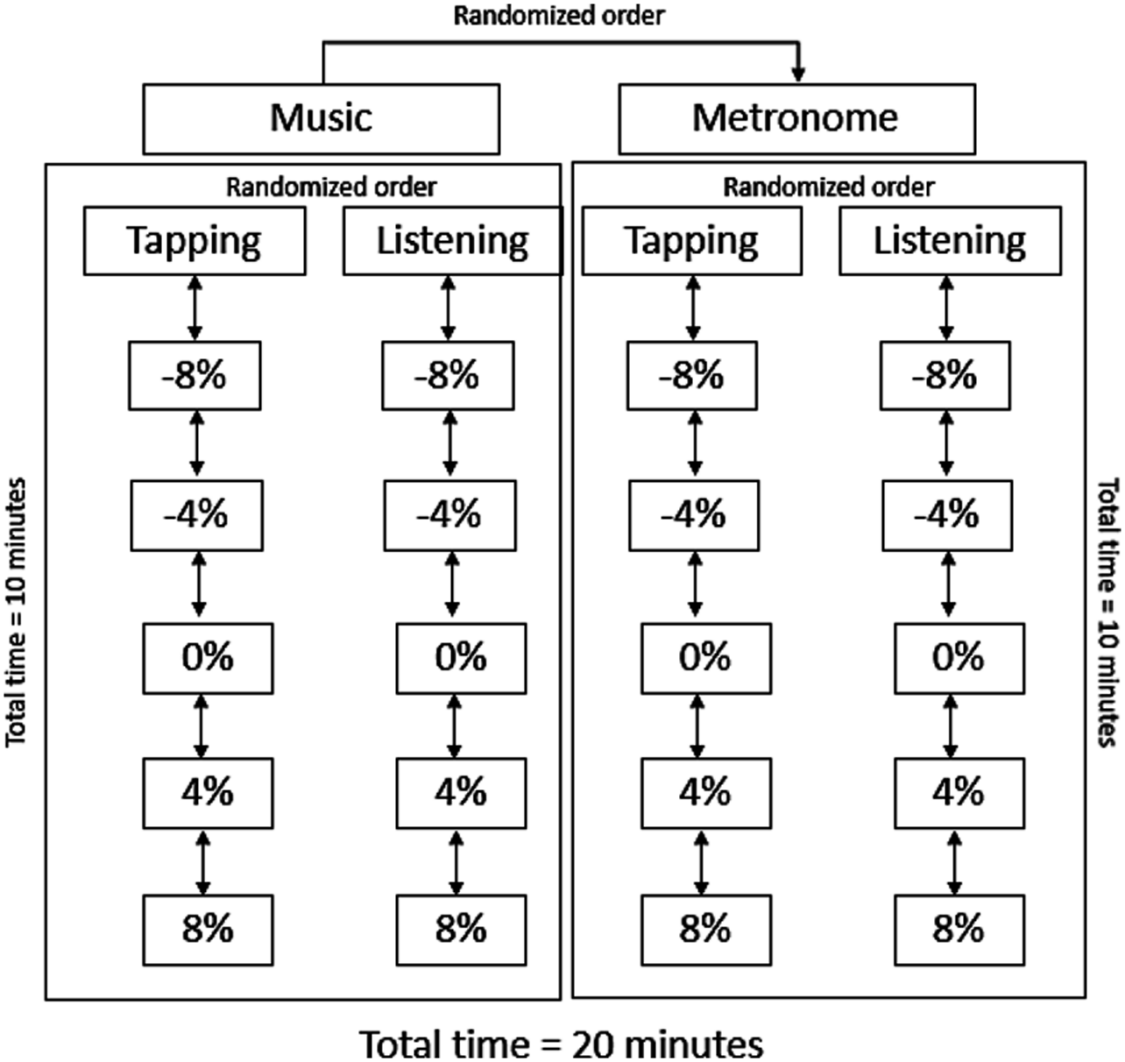
Overview of the experimental design.

For the tapping task, participants were provided with a custom-made tapping pad containing piezo sensors to detect tapping onsets. Before starting the experiment, participants familiarized with finger-tapping with the tapping pad, and they were asked to perform the tapping task with being as still as possible and without making sudden movements such as head turns (to account for the EEG recording). Participants were asked to perform two experimental tasks: tap on the tapping pad using their right index finger and listen to the beats in music and metronomes for one minute in a randomized order to five different tempi (preferred comfortable speed [(0%), −8, −4%, +4% and +8%]). This procedure was done in two blocks with a break in between, once with music, and once with metronomes in a randomized order. At the start of the experimental session, participants were asked to tap for 1 min at their comfortable tapping tempo. Thereafter, this was used to individualize the auditory tempi received per participant. For example, when participants tapped at a comfortable tempo of 100 taps per minute, 100 beats per minute (BPM) would be defined as there 0% tempo, 104 BPM for 4%, 108 BPM for 8%, 96 BPM for −4% and 92 BPM for −8%. Afterwards, participants were asked to tap and listen to music and metronomes in two separate randomized blocks. Within each block, five different tempi (−8%, −4%, 0%, 4%, 8%) were presented, where participants had to listen and tap to in a randomized order. Thus, in total, participants listened to 5 tempi and tapped to 5 tempi, both with music and metronomes. In total, the experimental conditions lasted for 20 min. On overview of the experimental condition can be found in Figure 2.

#### 2.3.1 Auditory stimuli

The auditory stimuli were delivered using the D-Jogger application (35), an interactive music player delivering the auditory stimuli and logging inter-onsets of the beats and taps in order to compute the synchronization outcome measures. For music specifically, the songs were categorized into six genres: disco, pop, soft pop, pop rock, instrumental, and variety from which the participant could choose one genre for the full experimental session. To clarify, participants tapped and listened to one genre of music, consisting different songs at different tempi, during the complete experiment. Songs for the different tempi were selected within the selected genre. The system selected a song/metronome that matched to each specific tempo and within a specific genre when tapping or listening to music. A familiarization task involving the song “Sanctum” by the artist “Shades of the Abyss” was administered to instruct participants to synchronize their taps with the beat of the music.

#### 2.3.2 Data acquisition

To record finger-tapping onsets, a Teensy 3.2 microcontroller operating as a serial/MIDI hub was used. When the participants started tapping, the Teensy microcontroller received input from the piezo sensors inside the tapping pad. Every time a beat in the music or metronomes was presented or a tap was recorded, a MIDI message was sent to the Teensy to log its timestamp on the serial port. Additionally, at the start of the first beat, a TTL trigger was sent to the EEG software to start the EEG recording using a BNC connection. To record the EEG data, the ANT-Neuro eego mylab system was used at a 1 kHz sampling rate. All impedances were kept below 20 kΩ. Data was referenced using the CPz electrode as reference electrode.

#### 2.3.3 Behavioral outcome measures

These outcome measures are related to synchronization consistency and accuracy. For a detailed overview please see (36).

##### 2.3.3.1 Relative phase angle (rPA)

De rPA is a measure of synchronization accuracy expressed in degrees and measures the timing of the tap relative to the beat. This can be either negative, tap before the beat, or positive, tap after the beat. The rPA was calculated using the following formula:


$$\phi =360*\left(\frac{T_{n}-B_{n}}{B_{n-1}-B_{n}}\right)$$

In this formula, T_n_ is the participant’s tap onset n and B_n_ is the onset of the closest beat.

##### 2.3.3.2 Resultant vector length (RVL)

The RVL is a measure of synchronization consistency ranging from 0 to 1 and measures the stability of the rPA over time. A steep distribution of the rPA’s over time results in a high RVL (maximum value of 1) and would indicate that all taps coincide with the beat. Conversely, a low RVL (minimum value of 0) suggests an unstable synchronization with a broad and multimodal distribution of the rPA over time. The following formula was used to calculate the RVL:


$$R=|\frac{1}{N}\sum_{n=1}^{N}e^{i\varphi T_{n}}|$$

where *R* is the resultant vector length, *N* is the total number of tap events, Tn is the onset time of the n-th tap, and φTn is the relative phase angle at that time.

##### 2.3.3.3 Mean asynchrony

The mean asynchrony consists of the mean difference between the participant’s tap onset and the closest beat in the metronome or music expressed in milliseconds.

##### 2.3.3.4 Inter-tap-interval (ITI)

The time between each tap expressed in seconds.

#### 2.3.4 Neural outcome measures

##### 2.3.4.1 Neurophysiological data

To compute the stability index, the pipeline proposed in the work of Rosso et al. (22) was used. A detailed explanation of this pipeline can be found there (22).

##### 2.3.4.2 Pre-processing of EEG data

For the pre-processing of the data a pipeline integrating functions from the Fieldtrip toolbox for Matlab were used (37). First, bad channels were identified by means of visual inspection of the raw time series data and variance distribution across channels. Then, the recordings were re-referenced to the average of all the electrodes after channel rejection. To remove slow drifts, a high-pass Butterworth filter with 0.5 Hz cut-off was applied to the raw recordings. A low-pass Butterworth filter with 45 Hz cut-off was applied to remove high-frequency muscular activity. Last, a notch filter was centered around 50 Hz to remove power-line noise up to the 3rd harmonic.

Independent component analysis (ICA) was conducted on full rank data to remove eye-blinks and eye-movement artifacts. This was done via visual inspection of the topographical maps and time series of the component’s activation. The ICA was done using the “runica” algorithm embedded in Fieldtrip, excluding the references electrode Cpz and all bad channel time series removed previously. The frontal components exhibiting the typical frontal distribution generated by eye-blinks and eye-movements were removed. After the ICA, data was inspected visually, especially for the electrodes where the activation of the artefactual component was maximal (channel F, AF and Fp clusters). Finally, rejected bad channels were reconstructed after artifact removal by computing a weighted average of all neighbors as implemented in Fieldtrip.

###### 2.3.4.2.1 Generalized eigendecomposition (GED)

**Figure 3:**
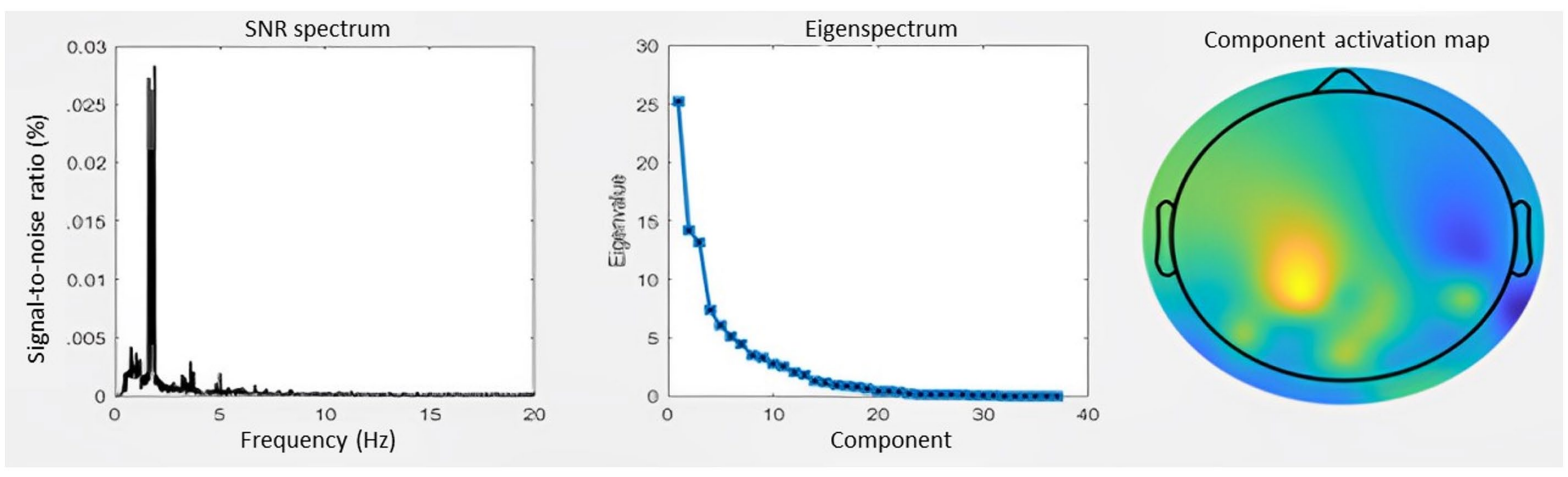
Generation of the entrained component. **(A)** SNR spectrum. The grand-average power spectrum is represented here in the percentage signal-to-noise ratio between each data point and the mean power in the neighboring bins (0.5 Hz) **(B)** Eigenspectrum. The grand-average eigenvalues sorted in descending order exhibit a steep exponential decay. **(C)** Topography. The grand-average coefficients of activation are shown in the topographic plot.

In order to extract the entrained component in the EEG signal and to avoid channel selection bias when optimizing the signal-to-noise ratio GED was applied as described in Rosso, Leman (22), (38, 39). This technique consists of a spatial filter to reduce the multivariate dataset to one dimension, here with the criteria of attunement to the stimulation frequency. For detailed overview of the GED method used we refer to Rosso, Leman (22). Last, the quality of the GED application was assessed by visually inspecting per participant the eigenspectrum, the topographical activation map and the power spectrum of the extracted oscillatory component. In total 10 PwPMS and 7 HCs were removed from the final dataset due to no correct extraction of one entrained component (Figure 3).

###### 2.3.4.2.2 Stability index

The entrained component was processed using a Gaussian filter (centered at 1.654 Hz with a 0.3 Hz bandwidth at half maximum) to extract accurate phase time series from the analytical signal. Thereafter, the analytical signal was calculated with the Hilbert transform and the instantaneous frequency time series were computed from the first derivative of the unwrapped phase angles time series (40). The derivative was then converted to Hz. A sliding moving median filter with a 400-sample window was applied to smooth the instantaneous frequency time series, reducing occasional extreme bursts caused by artificial activity that could distort the phase time series. Last, a standard deviation of this instantaneous frequency over the whole task was calculated, called the stability index. A high standard deviation indicates wide instantaneous frequency fluctuations, thus less stability of the entrained component. A standard deviation of 0 would indicate a perfectly stable oscillation.

#### 2.3.5 Statistical analysis

Descriptive data were tested for normality using a Shapiro–Wilk test. Subsequently, on normal distributed data, a t-test was performed. For non-normal distributed descriptive data, a Wilcoxon rank-sum test was performed. Before the analysis, all audio data were checked. All data containing errors were removed (*n* = 5). Behavioral data were analyzed using a Mixed-Model analysis by backward model building to the primary outcome measures (RVL, rPA, mean asynchrony and inter-tap-interval, stability index) with group (HCs vs. PwPMS) as between-subjects and tempi (5 conditions) and condition (music vs. metronome) as within-subjects variables and with each individual subject as random effects. The residuals of the models were checked for heterogeneity, and those not complying were exponentially transformed, which applied for RVL. A multiple comparisons Tukey’s test was further performed as a *post hoc* test when interactions were present. Additionally, spearman rank correlations (due to non-normal distributed data) between the stability index and RVL were performed. Last, a linear regression analysis was performed on the effect of disability score, motor impairments (EDSS and 9HPT) and cognitive functioning (COWAT, PASAT and SDMT) on synchronization consistency (RVL) as dependent variable.

## 3 Results

### 3.1 Participants

In total 19 PwPMS (mean age = 52.42, SD = 2.13) and 16 HCs (mean age = 56.70, SD = 2.32) were included in the study. Participants did not differ significantly in terms of education level and baseline tapping frequency (see Table 1). PwPMS did show impaired manual dexterity measured by the 9HPT (mean = 26.11 > 18, SD = 6.41) (41).

**Table 1:** Demographic and clinical characteristics of the study sample.

Descriptive information	PwPMS (*n* = 19)	HC (*n* = 16)	*t* Test (Prob > ltl)
Demographic			
Age (years)	52.42 ± 2.13	56.50 ± 2.32	ns
Gender (M/F)	13/6	6/10	ns
Education (years)	7.11 ± 0.57	8.63 ± 0.62	ns
EDSS (0–10)	4.24 ± 1.13	/	N/A
Motor functions			
9HPT right hand (s)	26.11 ± 6.41	/	N/A
9HPT left hand (s)	32.66 ± 32.84	/	
Baseline inter-tap-interval (median)	595.85 ± 94	607.38 ± 105.76	ns
Cognitive functions			
Buschke SRT (a.u.)	37.47 ± 2.11	21.25 ± 2.30	<0.0001
7 / 24 SRT (a.u.)	29.32 ± 0.95	31.13 ± 1.03	ns
COWAT	35.16 ± 1.96	35.75 ± 2.13	ns
PASAT (N)	36.42 ± 1.50	48.50 ± 1.63	<0.0001
SDMT (N)	50.89 ± 2.20	59.06 ± 2.39	0.0096
Stroop Color Test I (seconds)	56.68 ± 1.35	49.88 ± 1.47	0.0017
Stroop Color Test II (seconds)	67.16 ± 1.48	64.00 ± 1.61	ns
Stroop Color Test III (seconds)	97.68 ± 2.32	92.63 ± 2.53	0.0160
MBEA – Rhythm (15)	11.71 ± 1.95	11.53 ± 1.85	ns
Self-reported questionnaires			
BMRQ (100)	68.67 ± 9.89	/	N/A
HADS (42)	13.83 ± 7.43	/	N/A

In terms of cognitive testing, significant differences were observed in Buschke (*t* = 5.19, *p* = <0.0001), PASAT (*t* = −5.46, *p* = <0.0001), SDMT (*t* = −5.52, *p* < 0.01), Stroop Color Test I (seconds) (*t* = 3.42, *p* < 0.01), and Stroop Color Test III (seconds) (*t* = 1.47, *p* = 0.0160). When cognitive results were compared to normative data, an impaired score was found for PwPMS on the PASAT only (42), indicating impaired information processing speed and sustained divided attention. PwPMS overall experience reward when listening to music as measured by the BMRQ. Overall no indication of depression or anxiety was seen. Last, participants did not show impairments in terms of rhythm perception as assessed by the rhythm perception section of the MBEA (43).

### 3.2 Behavioral outcomes

#### 3.2.1 Relative phase angle

**Figure 4:**
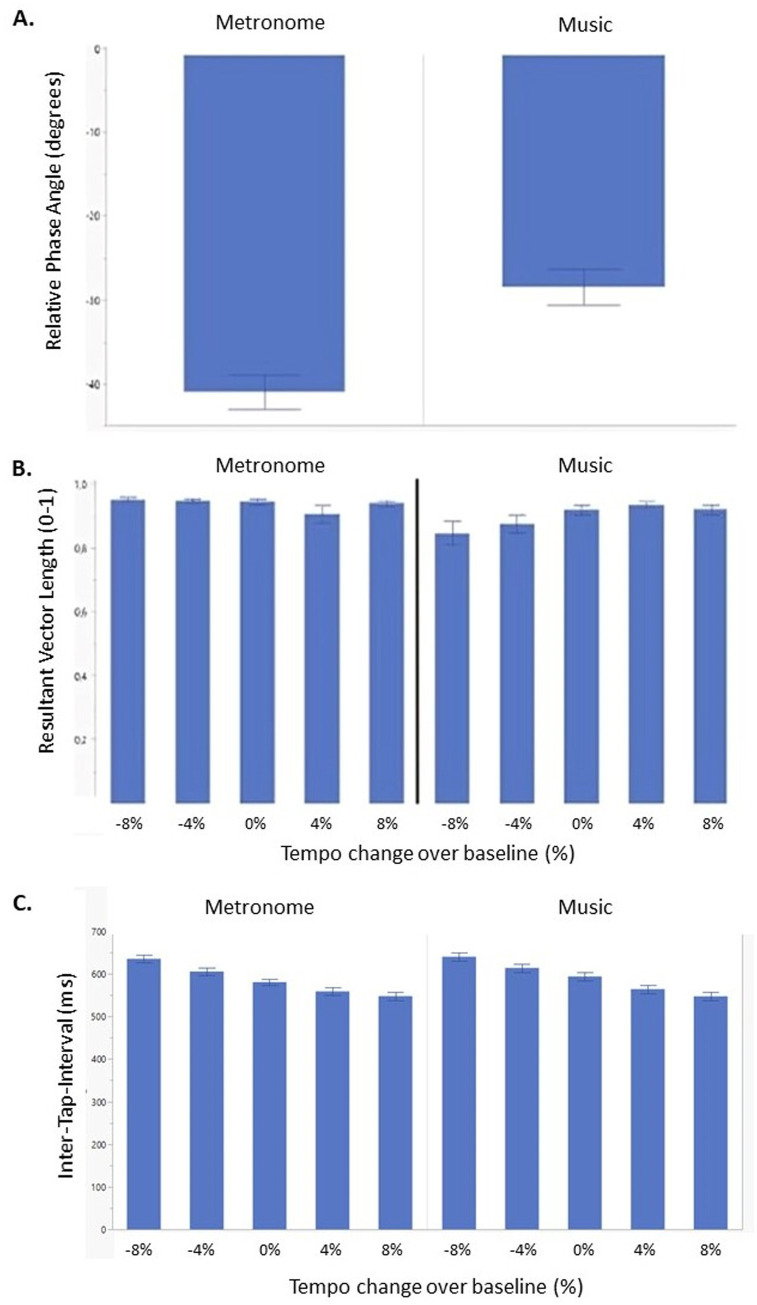
Synchronization consistency (RVL) **(A)**, relative phase angle **(B)** and Inter-Tap-Interval **(C)** of persons with progressive multiple sclerosis (PwPMS) and healthy controls (HC) tapping to metronome and music at different tempi, −8%, −4%, 0% and +4% and +8% of preferred tapping frequency. Mean standard errors are shown.

Overall, all participants anticipated the beat. A significant main effect was found for conditions (*F*(1, 298) = 26.42, *p* < 0.001) (Figure 4A), but no effect of tempo or group was found. *Post-hoc* analysis showed a significant higher rPA for metronomes (rPA = −40.20) compared to music (rPA = −30.92) (*t* = −5.14, *p* < 0.001).

#### 3.2.2 Resultant vector length

Results showed that overall, all participants were able to synchronize their taps to the beat for all tempi. A significant interaction effect was found between condition and tempo (*F*(4,295) = 3.28, *p* = 0.012) (Figure 4B). *Post-hoc* analysis showed higher consistent synchronization for negative tempi for metronome compared to music conditions (−8% vs. − 8%: *t* = 4.28, *p* < 0.001), (−8% vs. − 4%: *t* = 4.36, *p* < 0.001), and (−4% vs. − 8%: *t* = 4.02, *p* < 0.001), while no significant *post-hoc* results were found for positive tempi.

#### 3.2.3 Mean asynchrony

Results showed a main effect of condition (*F*(1,298) = 34.27, *p* < 0.0001). No interaction effects were found. *Post-hoc* analysis showed a higher mean asynchrony for metronome (mean asynchrony = −64.95 ms) compared to music (mean asynchrony = −42.96 ms) conditions (*t* = −5.85, *p* < 0.001).

#### 3.2.4 Inter-tap-interval

Results showed a significant main effect of tempo (*F*(4, 309) = 51.45, *p* < 0.001) and condition (*F*(1,309) = 8.10, *p* < 0.01) (Figure 4C). *Post-hoc* analysis indicated a higher median ITI for low compared to comfortable and high tempi. Additionally, *post-hoc* tests revealed higher ITI for metronome (median ITI = 602.25) compared to the music condition (median ITI = 589.39) (*t* = 2.85, *p* < 0.01).

No significant differences between groups was found for all behavioral outcome measures indicating no significant difference between HCs and PwPMS in terms of synchronization consistency and accuracy.

### 3.3 Neural outcomes

#### 3.3.1 Stability index

**Figure 5:**
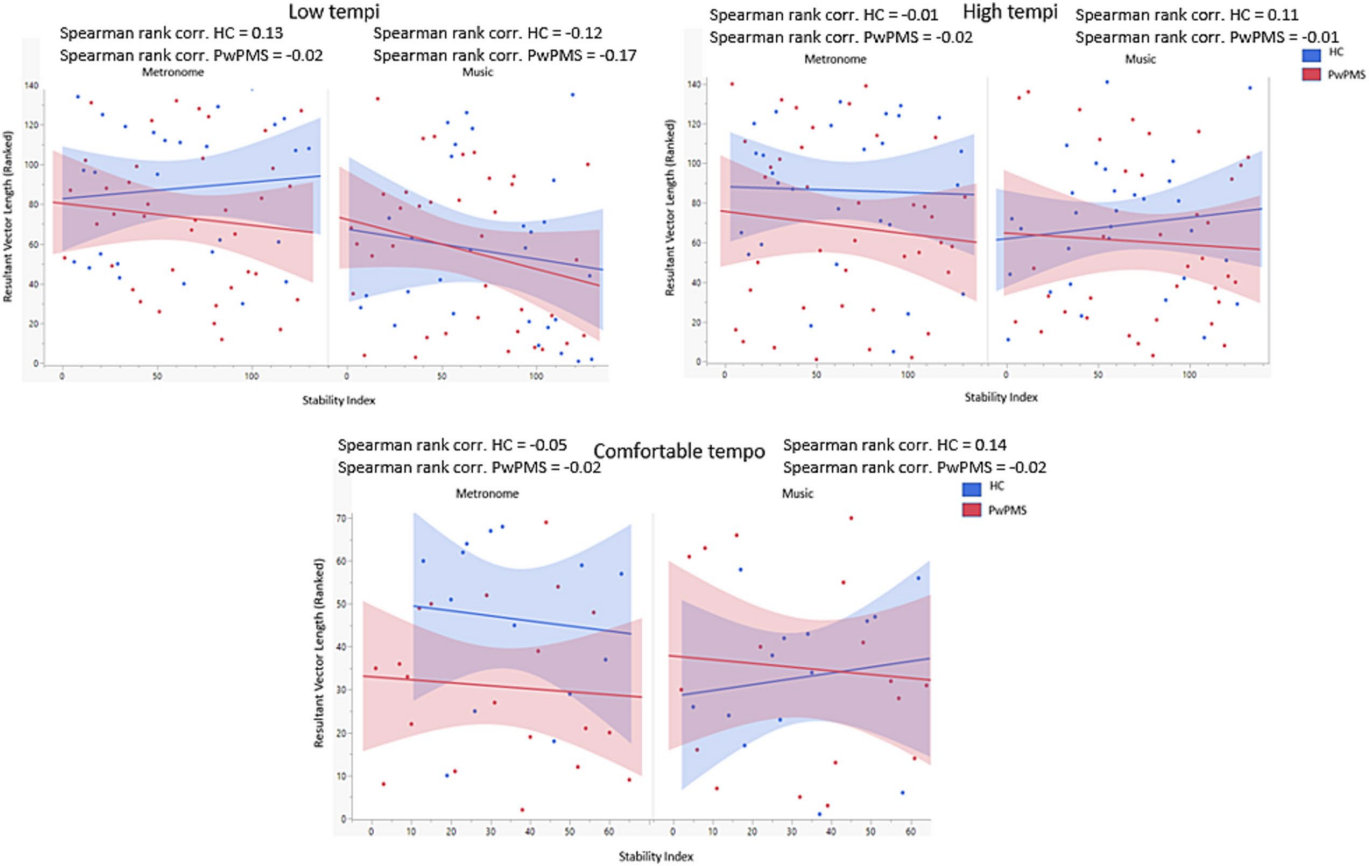
Correlation between synchronization consistency (RVL) and Stability index for low (−8% and −4%) comfortable (0%) and high (+4% and +8%) tempi of persons with progressive multiple sclerosis (PwPMS) and healthy controls (HC) tapping to metronome and music.

The mixed model analysis showed no significant main or interaction effects. Additionally, Spearman-Rank correlations were performed between synchronization consistency (RVL) and the stability index (Figure 5). RVL data were ranked due to a non-normal distribution of the data. No significant correlations were found. However, for HCs a trend can be seen for more consistent synchronization outcomes when a lower stability index (low standard deviation in the entrained component) was detected for music conditions for low tempi, while for metronome conditions for low, comfortable and high tempi. For PwPMS, this trend was observed for music conditions for low, comfortable and high tempi, and for metronome conditions for low, comfortable and high tempi.

### 3.4 Clinical factors impacting synchronization abilities

A regression analysis was performed with synchronization consistency (RVL) as dependent variable and 9HPT, EDSS, COWAT, PASAT and SDMT as factors for each condition (music and metronomes). A significant result was seen for EDSS (*p* = 0.024, RSquare = 0.13) and 9HPT (*p* < 0.001, RSquare = 0.28) for metronome conditions, indicating a higher synchronization consistency for better scores on the 9HPT and lower disability. For music conditions, significant results were found for COWAT (*p* = 0.005, RSquare = 0.07), PASAT (*p* < 0.001, RSquare = 0.25) and SDMT (*p* = 0.013, RSquare = 0.25), indicating higher synchronization consistency when higher scores on the COWAT and PASAT were seen.

## 4 Discussion

This study hypothesized a difference in synchronization consistency between healthy controls and PwPMS. However, contrary to our hypothesis, the results indicate preserved auditory-motor synchronization capacities for PwPMS even though some participants had higher disability scores (EDSS) and impaired manual dexterity. This could be explained by the spared perception and prediction capacities and the generation of an appropriate motor response, due to either preserved neural capacities or neural compensation strategies. In a previous study in pwMS, neural compensation was demonstrated by enhanced BOLD signal changes in the subcortical structures to cope with deteriorated spatial and temporal accuracy during a sustained finger motor task in order to re-establish adequate motor performance (13).

Our results are contradictory to other studies on auditory-motor finger-tapping showing significant differences between persons with MS and HCs (15). However, these differences may stem from the distinct experimental design, where in the study of Bonzano et al. (15) participants were required to perform more complex, coordinated, repetitive finger-tapping movements involving sequences in synchronization with an external cue, while wearing a sensor-equipped glove to measure motor performance. Moreover, no measure of synchronization consistency was reported as they only reported inter-tap-intervals. To our knowledge no other studies have investigated synchronization consistency during an auditory-motor coupling task in MS specifically. Additionally, studies on auditory-motor synchronization during walking have shown differences between HCs and PwMS (3). These differences, compared to those observed in the present study could be attributed to the underlying networks engaged during the two different movement tasks. Finger-tapping engages predominantly cortical brain regions, and it is also less physically demanding. While walking involves gross motor control and recruits both cortical and sub-cortical brain regions (44, 45). To elaborate, during walking, the mesencephalic locomotor region (MLR) in the brainstem plays a key role in locomotion by regulating muscle tone and rhythm generation, and then interacting with central pattern generators (CPGs) in the spinal cord, which generate the rhythmic, bilateral limb movements (33, 34). In contrast, these brainstem-spinal circuits are not engaged during unilateral finger tapping, as this movement is voluntary, consciously timed, and primarily controlled by cortical structures (46–48). Given that PwPMS involves widespread cortical and subcortical degeneration even more so compared to relapsing and remitting MS due to widespread demyelination (49), we hypothesized group differences to appear in the finger-tapping task as well. However, the lack of group differences during finger-tapping may reflect the simplicity and volitional control of the task, which may allow for functional compensation by intact cortical regions despite disease-related degeneration (50, 51). In contrast, walking, which engages distributed brain networks including the brainstem and spinal pattern generators (52), may reveal deficits that finger tapping does not. Future studies could incorporate a unilateral foot-tapping condition to further investigate how different types of movements contribute to auditory-motor synchronization in PwPMS. Like finger-tapping, unilateral foot-tapping is a voluntary, cortically driven movement that would not involve spinal pattern generator networks typically involved in locomotion (53). Including such a condition would allow for the investigation of how synchronization mechanisms may differ across motor tasks that vary in their neural control demands, offering further insight into the specific contributions of cortical and subcortical structures in auditory-motor processing in PwPMS. Contrary to our hypothesis, overall no differences between tempi were observed, indicating no effect of potential processing delays in PwPMS for low or high tempi. Increments of 4% were chosen as it has been shown that when tempo surpasses a preferred frequency by more than 4%, active cognitive control is required to maintain synchronization – albeit in the context of walking (23). While our hypothesis regarding increased synchronization difficulty at faster tempi was informed by findings from walking-based studies (e.g., Moumdjian, Moens (3)), it is noteworthy that finger tapping and walking rely on distinct neural mechanisms. As elaborated earlier, walking involves whole-body coordination, balance control, and subcortical locomotor networks (54, 55), whereas finger tapping is a cortically driven (53), low-effort movement involving minimal biomechanical and sensory-motor complexity. As such, cognitive control demands associated with synchronizing to faster tempos may differ between upper and lower limb movements. This distinction may explain why the synchronization differences reported in gait studies, such as Moumdjian, Moens (3), were not observed in our study using a finger-tapping task.

However, our results could indicate a preserved neural reserve for simple finger tapping and spared auditory-motor synchronization capacities and compensation strategies to overcome potential challenges in terms of motor control for high and low tempi during finger-tapping. In hindsight, this may be linked to the limited capacity for motor execution in our task, while auditory-motor coupling is preserved. Our results show higher synchronization consistency for metronomes compared to music conditions for both groups, which is consistent with previous studies using synchronization tasks (12, 56). Specifically, in previous studies involving walking, it has been shown in both healthy individuals and persons with relapsing remitting MS that synchronization consistency to metronomes is higher compared to music. This due to the complex rhythmic structure seen in music (57), indicating that perceiving and extracting rhythmic patterns is more challenging with music than with unambiguous isochronous metronome beats. This suggests that individuals with weaker rhythm perception may find it more challenging to synchronize their movements to music as studies have indicated that poorer rhythm perception abilities result in poorer synchronization capacities (58). In this study, to assess rhythm perception, participants completed the rhythm perception section of the MBEA test, and results demonstrated that both groups had intact rhythm perception abilities. This due to the complex rhythmic structure seen in music (57), indicating that perceiving and extracting rhythmic patterns is more challenging with music than with simple metronome beats. This suggests that individuals with weaker rhythm perception may find it more challenging to synchronize their movements to music as studies have indicated that poorer rhythm perception abilities result in poorer synchronization capacities (58). All participants anticipated the beat as typically reported in finger-tapping synchronization tasks (59), but tapped closer to the beat for music compared to metronome conditions. However, mean negative asynchronies for both conditions were below -100 ms suggest adequate anticipation (60). Evidence shows a higher mean asynchrony for metronomes compared to music using a finger-tapping task (61). An explanation for the above could stem from the clear pulses provided in metronome rhythms resulting in clear central representations and thus anticipation of the beat. Complex structures seen in music can result in the generation of more positive asynchronies, due to the process of error-correction, complicating the generation of an internal model of the rhythmic structure. Additionally, participants were not able to choose specific songs of their liking, however, they were asked to select a genre. We do acknowledge that song familiarity could potentially influence beat perception and prediction abilities However, to account for this bias, song selection was purely based on the closeness of the selected tempo of the experimental condition.

The challenge of anticipating in presence of rhythmic complexity could have contributed to an additional cognitive load (59), potentially explaining the correlation between cognition and synchronization consistency in music in PwPMS. The effect of cognition on key tapping has recently been shown in persons with dementia showing a trade-off in tapping frequency and speed during fast key tapping between persons with and without high levels cognitive impairment (62). Specific for PwPMS, impairments in information processing speed are seen due to widespread demyelination and loss of neural connectivity. The complexity in the musical structure could impose more cognitive demands, potentially surpassing the capacity of PwPMS with cognitive impairment in terms of forming and maintaining temporal predictions.

While our results did not show significant differences in terms of synchronization consistency between both groups, clinical factors in PwPMS correlate with synchronization consistency. When tapping to a metronome, disability level and manual dexterity impacts synchronization consistency as previously shown by Bonzano, Pardini (13) indicating the effect of upper limb impairment on a pacing tapping task. The specificity of the metronome could be the task-related context as metronomes impose faster corrections (63) possibly making it more difficult for PwPMS with higher disability, due to loss of neural reserve, to compensate. Although no significant correlations were found for neural entrainment, a negative trend was observable in PwPMS for all tempi in both conditions (music and metronome), indicating higher synchronization consistency when less variability in neural entrainment was seen. However, this trend was not seen for HCs for low tempi for metronome conditions and for comfortable and high tempi for music conditions. The lack of significant effects for the stability index measure can be related to methodological issues. First, one could argue that the duration of the recording was too short in order to detect a stable oscillatory component resulting in a low signal-to-noise ratio over time. In comparison, the study of Rosso, Leman (22) measured neural entrainment during a 6.5 min tapping task and were able to extract an entrained component over time for all participants. Second, different tempi were applied for each participant based on each individual baseline comfortable tapping frequency. Therefore, to complete the GED analysis, the filter was individualized to different auditory frequencies, unlike the approach by of Rosso et al. (22) where one filter was applied to the standardized tempo. This may have impacted our results to some extent.

Our results have clinical implications, given the preserved auditory-motor synchronization capacities across a spectrum of impairment levels in individuals with MS. As a result, auditory-motor synchronization tasks can be applied to support upper limb training in PwPMS, who show often impairments in manual dexterity (64). To address manual dexterity and introduce greater challenge, more complex coordinated finger-tapping tasks such as in-hand sequential between-finger tapping, could be applied. However, our results also indicate an impact of cognitive impairment on synchronization consistency for music and the impact of disability score and manual dexterity on synchronization consistency for metronome conditions. Performing complex finger-tapping sequences require greater cognitive engagement, and therefore applicability of auditory-motor coupling may be different in persons with manifest cognitive deficits (15). Next, our results support opportunities to train temporal prediction skills, advancing from simple (here metronomes) to complex (here music) rhythms, and from comfortable pace to higher and lower tempi. Motor training at different tempi provide a rich spectrum for motor control challenges. Last, there are also potential applications in improving auditory-motor strategies to use for the rehabilitation of other motor functions such as for walking. Although, here, it is important to note that when employing auditory-motor synchronization strategies for walking rehabilitation, motor factors like reduced balance control or muscle weakness may reduce the synchronization consistency and related rehabilitation potential.

## 5 Conclusion

Spared auditory-motor synchronization abilities were observed in PwPMS when tapping to either music or metronomes. Cognition, manual dexterity and overall disability level impacted synchronization consistency to some extent. The results are supporting the potential use of auditory-motor coupling methodologies for the rehabilitation of motor impairments in PwPMS.

## Data Availability

The raw data supporting the conclusions of this article will be made available by the authors, without undue reservation.
